# Augmentation of Autoantibodies by *Helicobacter pylori* in Parkinson’s Disease Patients May Be Linked to Greater Severity

**DOI:** 10.1371/journal.pone.0153725

**Published:** 2016-04-21

**Authors:** Gunasekaran Suwarnalata, Ai Huey Tan, Hidayah Isa, Ranganath Gudimella, Arif Anwar, Mun Fai Loke, Sanjiv Mahadeva, Shen-Yang Lim, Jamuna Vadivelu

**Affiliations:** 1 Department of Medical Microbiology, Faculty of Medicine, University of Malaya, Kuala Lumpur, Malaysia; 2 Division of Neurology and the Mah Pooi Soo & Tan Chin Nam Centre for Parkinson’s & Related Disorders, University of Malaya, Kuala Lumpur, Malaysia; 3 Sengenics Sdn Bhd, University of Malaya, Kuala Lumpur, Malaysia; 4 Department of Medicine, Faculty of Medicine, University of Malaya, Kuala Lumpur, Malaysia; Aarhus University, DENMARK

## Abstract

Parkinson's disease (PD) is the second most common chronic and progressive neurodegenerative disorder. Its etiology remains elusive and at present only symptomatic treatments exists. *Helicobacter pylori* chronically colonizes the gastric mucosa of more than half of the global human population. Interestingly, *H*. *pylori* positivity has been found to be associated with greater of PD motor severity. In order to investigate the underlying cause of this association, the Sengenics Immunome protein array, which enables simultaneous screening for autoantibodies against 1636 human proteins, was used to screen the serum of 30 *H*. *pylori*-seropositive PD patients (case) and 30 age- and gender-matched *H*. *pylori*-seronegative PD patients (control) in this study. In total, 13 significant autoantibodies were identified and ranked, with 8 up-regulated and 5 down-regulated in the case group. Among autoantibodies found to be elevated in *H*. *pylori*-seropositive PD were included antibodies that recognize Nuclear factor I subtype A (NFIA), Platelet-derived growth factor B (PDGFB) and Eukaryotic translation initiation factor 4A3 (eIFA3). The presence of elevated autoantibodies against proteins essential for normal neurological functions suggest that immunomodulatory properties of *H*. *pylori* may explain the association between *H*. *pylori* positivity and greater PD motor severity.

## Introduction

*Helicobacter pylori* is a Gram-negative bacterium that chronically colonizes the stomach and duodenal lining of more than 50% of the human population worldwide [1]. Colonization may occur during childhood and tends to persist for life unless treated [2]. It is well established that *H*. *pylori* colonization increases the risk of gastroduodenal diseases, including peptic ulcers and gastric cancer [3]. In addition, the bacterium may influence the occurrence and progression of several extragastric diseases through the production of a low-grade inflammatory state, induction of molecular mimicry mechanisms, and interference with absorption of nutrients and drugs [4]. *H*. *pylori* has been associated with a variety of autoimmune disorders. Although *H*. *pylori* colonization takes place mainly in the antrum, *H*. *pylori*-driven autoimmune process causes gastric corpus atrophy [5]. Autoimmune gastritis, a silent and highly prevalent disease that only becomes clinically manifested with progression to corpus atrophy and development of iron deficient or B12-deficient (pernicious) anemia, is associated with autoimmune thyroiditis and type 1 diabetes mellitus [6]. An elevation of Th1-immune response against *H*. *pylori* Heat shock protein 60 (Hsp60) and an increment of transendothelial migration of T-cells may be linked to the development of atherosclerotic lesions in mice [7]. Seropositivity to *H*. *pylori* has been linked to the presence of anti-nuclear antibodies (ANA), anti-dsDNA, anti-Ro and some thrombophilia-associated antibodies, as well as negative associations with gastrointestinal-associated antibodies [8].

It was already reported in 1965 that peptic ulcers were more common among patients with Parkinson’s disease (PD) and more than 80% of these ulcers were found to precede parkinsonian symptoms by a mean of 8 to 10 years [9,10]. This was before the relationship of *H*. *pylori* to gastric pathology was discovered, but opened the way for suggestion and presentation of evidence that chronic *H*. *pylori* colonization and autoimmunity can contribute to PD [11,12]. Consistent with the earlier findings, a large population-based study found that prescriptions for eradication treatment for *H*. *pylori* colonization and proton pump inhibitors were associated with a 45% and 23% increased risk, respectively, of developing PD five or more years later [13]. Interestingly, we recently found that *H*. *pylori* positivity was independently associated with greater PD motor severity, even after controlling for the effects of age, PD duration and small intestinal bowel overgrowth status on motor function [14]. This is in line with objectively measured brady/hypokinesia and flexor-rigidity being worse and higher circulating natural killer cell count noted with *H*. *pylori*-positivity, over and above that can be explained by magnitude equivalent to that of a levodopa challenge [15,16]. Several other studies have suggested that *H*. *pylori* eradication may improve motor fluctuations in PD by improving levodopa bioavailability [17,18]. However, levodopa absorption is a ‘red herring’, since in a randomized controlled trial (where receipt of levodopa was an exclusion), *H*. *pylori* eradication alone reduced hypokinesia of gait in PD [19]. Furthermore, longitudinal observation showed that improved hypokinesia was specific to *H*. *pylori* eradication and antimicrobials for other indications did not improve hypokinesia [20].

Current indicative evidences on immune relationship of microbiome, *H*. *pylori* in particular, to PD has recently been reviewed [21]. There is a growing recognition that the gastrointestinal tract, which represents a vulnerable port of entry for pathogens, plays an important role in the pathogenesis of PD [22–24] and the sequelae of neuroinflammatory process induced by these pathogens has been described in both human and animal models of PD [25–28] Thus, to better understand the relationship between *H*. *pylori* and PD pathogenesis, host-pathogen interaction and host immune response, a preliminary study using an autoantigen array was performed to characterize the autoantibody repertoire of *H*. *pylori*-seronegative (control) and -seropositive (case) PD patients.

**Fig 1:**
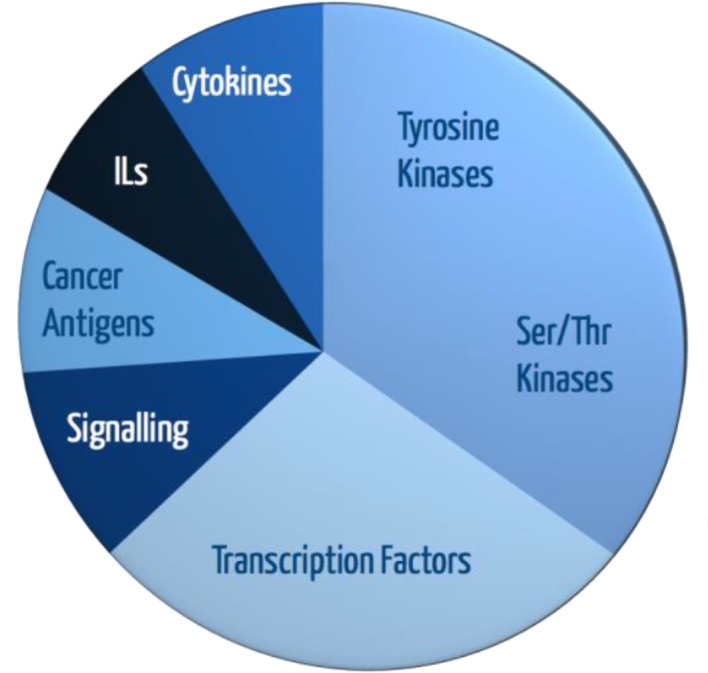
Stratification of 1636 immobilized proteins on array based on biological function.

The Sengenics Immunome (formerly Oxford Gene Technology's Discovery Protein Array) consists of 1636 immobilized full-length and correctly-folded proteins that represent different classes and various types of proteins, such as kinases and transcription factors (Fig 1). These proteins have been selected on the basis of being involved in the immune response. The proteins are immobilized on the array via an affinity tag, specifically the biotin carboxyl carrier protein (BCCP) domain of the *Escherichia coli* acetyl CoA carboxylase, reducing the possibility of affecting protein folding and function. Hence, each protein is expressed in insect cells as a fusion protein with a proprietary BCCP tag that monitors correct folding and minimizes non-specific binding [29]. Autoantibodies are produced by the immune system in many pathogenic processes. Since the appearance of autoantibodies may precede disease symptoms by many years and, due to the inherent amplification of the immune system, this array offers a powerful method in elucidating autoimmune diseases, as well as autoimmunity, involvement in the disease processes. The platform utilizes correctly folded proteins that have the ability to display native, discontinuous epitopes for the identification of specific autoantibody markers that may represent different functional processes (Fig 1) [29]. In addition, proteomic autoantibody profiling also provides a platform for recognizing and defining the signature serum autoantibodies of a disease, some of which may be potential biomarkers for PD [30].

## Materials and Methods

### Sample Collection

Blood samples were obtained from consecutive patients attending the University of Malaya Medical Centre Neurology Clinic who had a clinical diagnosis of PD assigned by a movement disorders neurologist (SYL) according to the Queen Square Brain Bank criteria. PD patients with a broad spectrum of disease severity were recruited and patients who underwent functional neurosurgery were excluded. The study received ethics approval from the Medical Ethics Committee, University of Malaya Medical Centre and written informed consent was obtained from all patients. All experiments conformed to the principles set out in the World Medical Association Declaration of Helsinki. Demographic and clinical data, including age of onset, PD duration, disease duration, Hoehn & Yahr staging [31] and daily levodopa equivalent units (LEU) were recorded. Blood samples were stored at -80°C before analysis.

### *H*. *pylori* Serology

Serological tests for *H*. *pylori* whole-cell antigen (WC) and cytotoxin-associated gene product A (CagA) were performed by enzyme-linked immunoassays (ELISA) using assays developed and validated by New York University as previously described [32]. Out of the 140 serum samples, 30 *H*. *pylori*-seropositive PD samples were designated as case and 30 age- and gender-matched *H*. *pylori*-seronegative PD samples were selected from the remaining cohort to serve as controls.

### Sengenics Immunome

Each critical experimental step of running the protein array was thoroughly checked by a second trained person who precisely recorded and cross-checked all steps in the protocol. This step was important to reduce operator bias. Samples were randomized. These samples were then stored at -20°C until the experimental setup was complete.

### Serum/Plasma Dilution

Samples were allowed to thaw at 20°C for 30 minutes. When completely thawed, each sample was vortexed vigorously and spun down for 3 minutes at 13,000 rpm using a microcentrifuge. After centrifugation, 22.5 μl of the sample was pipetted into 4.5 ml of Serum Assay Buffer (SAB) containing 0.1% v/v Triton, 0.1% w/v bovine serum albumin (BSA) in phosphate buffered saline (PBS; 20°C) and vortexed to mix. The tube was tilted during aspiration to ensure that the sera was sampled from below the lipid layer at the top but did not touch the bottom of the tube in case of presence of any sediment.

The array was removed from the storage buffer, placed into slide box containing 200 ml cold SAB and shaken on an orbital shaker at 50 rpm, for 5 minutes. When the slides have completed washing, the slide was placed, array side up, in a slide hybridization chamber with individual sera which had been diluted earlier. All slides were incubated on a horizontal shaker at 50 rpm for 2 hours at 20°C. The protein array slide was then rinsed twice in individual “Pap jars” with 30 ml SAB, followed by 200 ml of SAB buffer in the slide staining box for 20 minutes on the shaker at 50 rpm at room temperature.

Binding of IgG was detected by incubation with Cy3-rabbit anti-human IgG (Dako Cytomation) labeled according to the manufacturer's recommended protocols (GE Healthcare). Arrays were immersed in hybridization solution containing a mixture of Cy3- rabbit antihuman IgG solution diluted 1:1000 in SAB buffer for 2 hours at 50 rpm in 20°C. After incubation, the slide was dipped in 200 ml of SAB buffer, 3 times for 5 minutes at 50 rpm at room temperature. Excess buffer was removed by immersing the slide in 200 ml of pure water for a few minutes. Slides were then dried for 2 min at 240g at room temperature.

Hybridization signals were measured with a microarray laser scanner (Agilent Scanner) at 10μm resolution. Fluorescence levels were detected according to the manufacturer's instructions. Data sorting and analysis are done by customized computer scripts written using a Linux operating system, whereas each spot is plotted using Agilent Feature Extraction software.

### Statistical Analysis

The output from the microarray scanner is a raw.tiff format image file. In order to identify and detect the spots automatically and accurately, the GenePix Pro 7 software was used for spot segmentation. The objective of the spot segmentation is to perform a semi-automatic QC process in order to produce a viable result.

The main objectives of the statistical analysis are to determine the quality of the data, success rate of the experiment based on positive controls and statistically identifying putative biomarkers from the study. Data mining and analysis for both quality control and identification of biomarkers were done using customized scripts created in R and Perl.

Different methods of quality control based on both raw and normalized data were done to verify the quality of the protein array data before proceeding with the data analysis:

1 Median of the raw signal intensities were calculated from quadruplet protein spots on each slide (i.e. each sample):
  $$X=\overset{\;\;\;˜}{x}(m1+m2+m3+m4)$$ Equation 1
  m = signal intensity of replicates for each protein
  *X* = raw median for each protein in each sample
2 Median background signals were subtracted from the median raw median signal intensities.
3 Signal intensities of two positive controls (IgG and Cy3BSA) were examined.
4 Quantile normalization of data was performed with the exclusion of control proteins, i.e. normalization of only 1631 protein spots across all samples.

X=p×N Equation 2

Set *d* = 1√N,…..,1√N

Sort each column of *X* to give *X*_*sort*_

Project each row of *X*_*sort*_ onto *d* to get *X*′_*sort*_

Get *X*_*norm*_ by rearranging each column of *X*′_*sort*_ to have the same ordering as original *X*

*p* = number of proteins

*N* = number of samples

5 Percentage of coefficient of variant (CV%) of intra-protein, intra-slide and inter-array were calculated to determine the variations between the quadrupled signal intensity for each protein spot on the slide.

$$CV\%=\frac{M.A.D.}{Median}\times 100\%$$ Equation 3

*M*.*A*.*D*. = median absolute deviation of each sample

*Median* = median of quadrupled signal intensity of each protein

Identification and ranking of protein biomarkers were done using penetrance-based fold change. A penetrance-based fold change measures the likelihood that a given raw fold change (FC) is true, thus increasing the significance and reliability of the results. A step-by-step description of this method is as follows:

1 Quantile normalization of data was performed with the exclusion of control proteins, i.e. normalization of only 1631 protein spots across all samples as described in Eq 2.
2 Individual fold changes for both case and control were calculated by dividing each normalized data, *H* from Eq 2 by the mean of each protein across all samples *<P>*.

$${FC}_{Case}=\frac{H_{Case}}{<p>}$$ Equation 4

$${FC}_{Control}=\frac{H_{Control}}{<p>}$$ Equation 5

3 Penetrance frequency for both case (*Frequency*_*Case*_) and control (*Frequency*_*Control*_) were calculated for each protein.

$${Frequency}_{Case}=n\left({FC}_{Case}\geq 2\right)$$ Equation 6

$${Frequency}_{Control}=n\left({FC}_{Control}\geq 2\right)$$ Equation 7

4 Penetrance Fold Change for both case and control were calculated for each protein.

$$\begin{array}{c} Penetrance\;fold\;change_{Case}=\frac{\mu \left(H_{Case}\left[i\right]\right)}{\mu \left(H_{Control}\right)}\\ \left[i\right]=H_{Case}with\;FC_{Case}\geq 2 \end{array}$$ Equation 8

$$\begin{array}{c} Penetrance\;fold\;change_{Control}=\frac{\mu \left(H_{Control}\left[i\right]\right)}{\mu \left(H_{Case}\right)}\\ \left[i\right]=H_{Control}with\;FC_{Control}\geq 2 \end{array}$$ Equation 9

A volcano plot was achieved by calculating the p-value using a Student T-Test for the two groups and plotting it against the Log2 transformed overall fold change (ratio). The overall fold change was calculated by dividing the mean of each protein across all case samples, μ(*H*_*Case*_) with the mean of each protein across all control samples, μ(*H*_*Control*_).

$$Overall\;fold\;change=\frac{\mu \left(H_{Case}\right)}{\mu \left(H_{Control}\right)}$$ Equation 10

Biomarkers were identified and ranked according to the following criteria: (1) p-value < 0.05; (2) for up-regulated biomarkers, Penetrance fold change difference (i.e. *Penetrance* f*old* c*hange*_*Case*_− *Penetrance* f*old* c*hange*_*Control*_) must be ≥ 2 and Frequency Differential ≥ 1; (3)

for down-regulated biomarkers, Penetrance fold change difference (i.e. *Penetrance* f*old* c*hange*_*Case*_ − *Penetrance* f*old* c*hange*_*Control*_) must be ≤ -2 and Frequency differential ≤ -1; (4)

frequency percentage in case (i.e. *Frequency*_*Case*_/*Number of* c*ase* × 100%) must be ≥ 10%; and (5) frequency percentage in control (i.e. *Frequency*_*Control*_/*Number of* c*ontrol* × 100%) must be ≥ 10%.

## Results

Out of 140 (71.4% Chinese, 14.3% Malay and 14.3% Indian) PD patients recruited, 67 (47.9%) were found to be *H*. *pylori* seropositive. Distribution of *H*. *pylori*-seropositive patients by racial group was 44.0% Chinese, 50.0% Malay and 65% Indian. Among PD patients who were *H*. *pylori* seropositive, 30 were selected for this study. The control group consisted of 30 age- and gender-matched *H*. *pylori*-seronegative PD patients selected from the same cohort. All subjects were Chinese with no significant differences in age, gender, age of PD onset, disease duration, disease severity (Hoehn & Yahr stage), and medication requirements (total levodopa equivalent units) between the two groups (Table 1).

**Table 1:** Demographics, clinical presentation and medication of *H*. *pylori*-seronegative (control) and *H*. *pylori*-seropositive (case) PD subjects.

	Case (N = 29)	Control (N = 29)	p Value
**Race**	Chinese	Chinese	
**Gender (% Male)**	44.8	55.2	0.600
**Age (years)**	65.3 ± 7.2	63.6 ±9. a	0.318
**Age of diagnosis (years)**	57.4 ± 9.9	56.6 ± 9.7	0.703
**Disease duration (years)**	7.9 ± 5.3	7.0 ± 6.8	0.581
**Hoehn & Yahr score**	2.45 ± 0.8	2.52 ± 0.9	0.767
**Total LEU (mg/day)**	608.0 ± 457.1	454.0 ± 298.5	0.213

LEU = levodopa equivalent units.

### Quality Control (QC)

Quality control of the raw signal intensities shows good array quality and consistent data acquisition (S1–S4 Figs).

### Analysis of IgG Control

**Fig 2:**
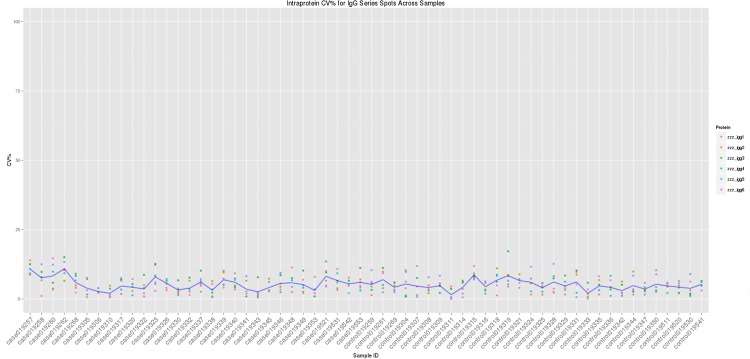
Intra-protein, intra-slide and inter-array percentage coefficient of variation (CV%) of 6 IgG controls across all samples. The blue line represents the mean CV% for each sample.

Percentage of coefficient of variation (CV%) between the arrays on each slide determines the quality of the spot intensities and also allows us to judge the variability of the IgG control spots in each sample. In this study, the mean CV% for the IgG control spots across all 60 samples was calculated to be 5.23% (Fig 2). The QC cutoff is 10%.

**Fig 3:**
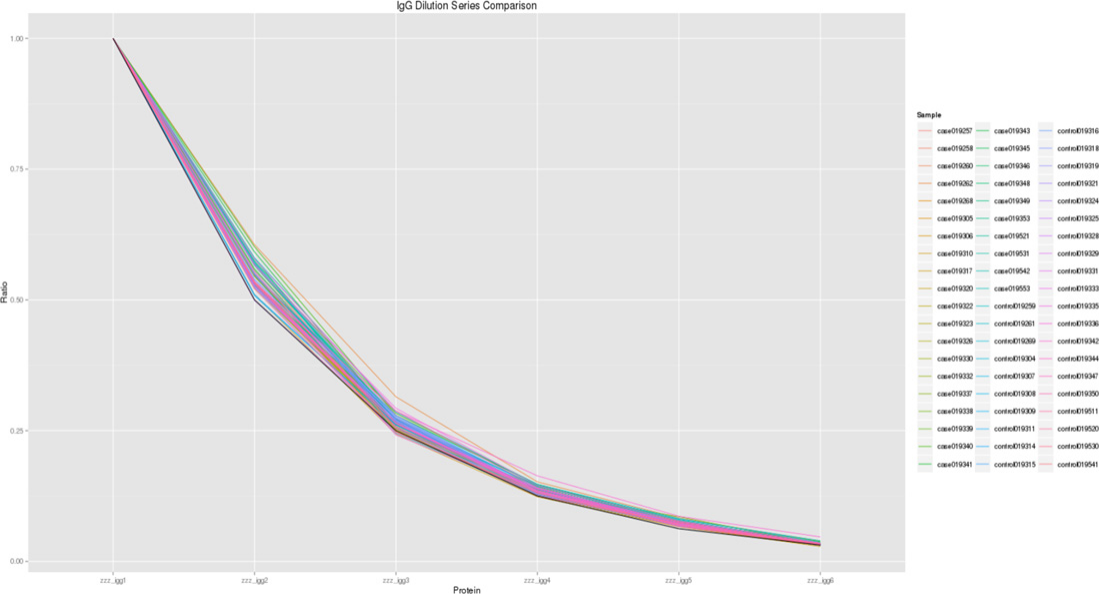
Comparison of IgG dilution series of all 60 samples. The experimental (ideal) IgG dilution series is plotted as a black line.

Variation studies of IgG serial dilution to the experimental (ideal) IgG serial dilution can be seen in Fig 3. A ratio of the median relative fluorescent units (RFU) of the IgG1 to IgG6 for each sample (IgG1:IgG2:IgG3:IgG4:IgG5:IgG6 = 1:0.5x:0.25x:0.125x:0.0625x:0.03125x) was done and plotted against the IgG dilution series. The mean standard deviation for the 60 samples to the experimental (ideal) IgG serial dilution was calculated to be 0.0117.

### Analysis of Cy3-BSA Control

**Fig 4:**
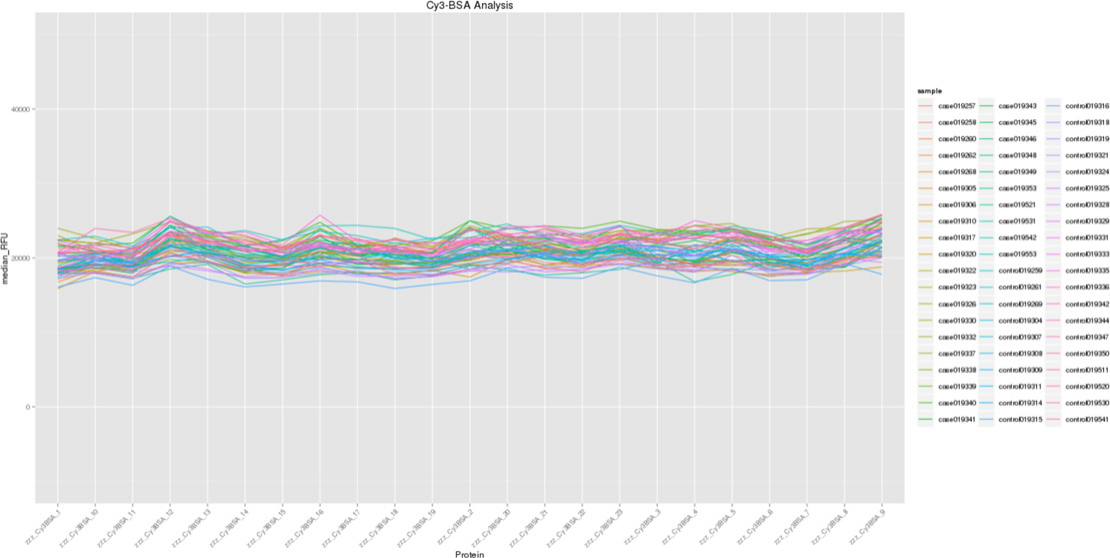
Line plot of controls Cy3-BSA from this protein array experiment.

Cy3-BSA controls act as positive controls for each array on the slide. Cy3-BSA markers were present on each slide and their concentrations were kept constant throughout the experiment. Fig 4 shows the median RFU of Cy3-BSA1 to Cys-BSA23 for all 60 samples. The figure reflects successful calibration of RFU from this protein array experiment.

### Intra-Protein, Intra-Slide and Inter-Array CV%

**Fig 5:**
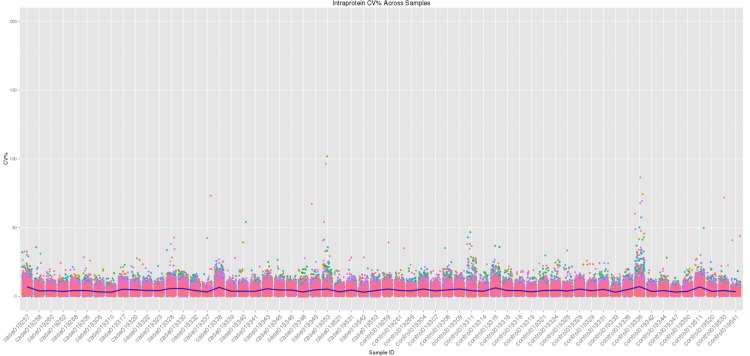
Intra-protein, intra-slide and inter-array CV% for all 60 samples. Samples with proteins presenting high intra-protein CV% were due to background noise in one or two replicates in those sample slides. However, for analysis purposes, the median values were used for each protein (Eq 1) therefore removing any outliers (i.e. high or low values).

Coefficient of variation between the arrays on each slide determines the quality of the spot intensities and also allows us to judge the variability of the protein and control spots on each slide. The threshold for CV% is < 10%. Fig 5 shows the intra-protein, intra-slide and inter-array CV% for all samples. The mean CV% for all samples was calculated to be 4.50%.

### Antigens with High Autoantibody Reactivity

**Fig 6:**
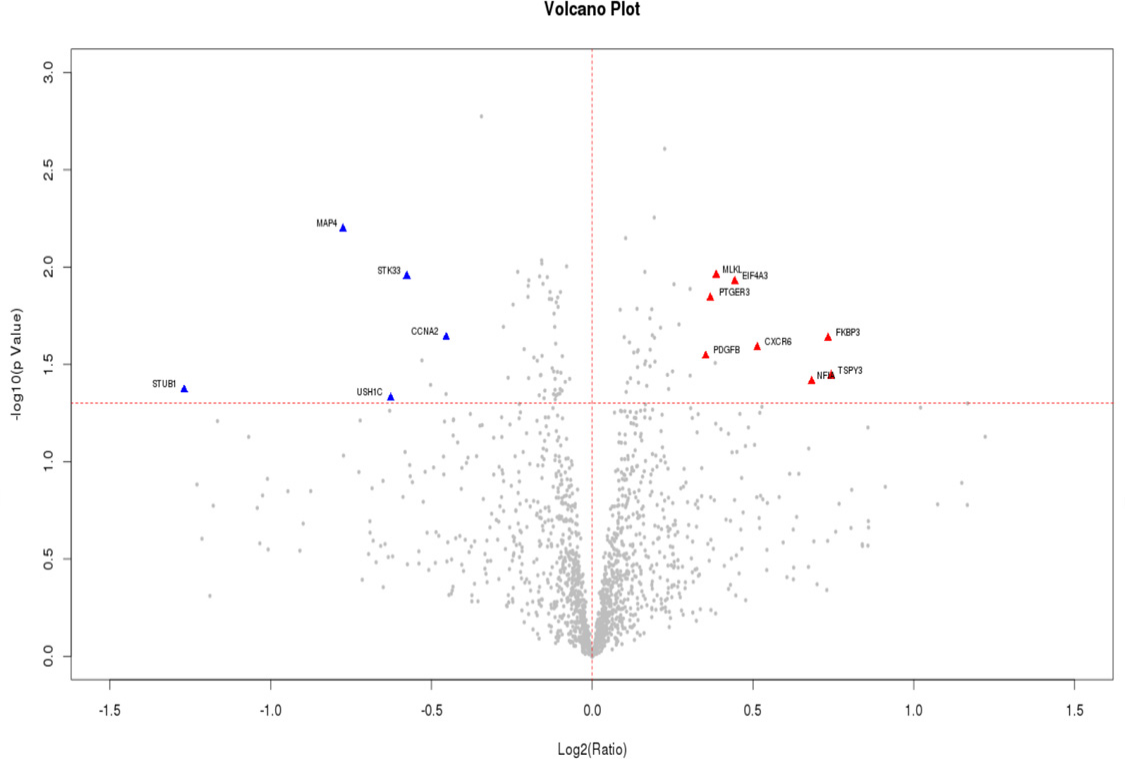
Volcano plot depicting both up-regulated and down-regulated autoantibodies identified using the penetrance-based analysis.

Significant autoantibodies were identified and ranked using two methods: penetrance-based threshold (Tables 2 and 3) and volcano plot analysis (Fig 6). Using the Sengenics Immunome protein array, which consists of 1636 immobilized full length and correctly folded proteins as bait, 13 significant autoantibodies were identified and ranked with 8 up-regulated and 5 down-regulated in the case group.

**Table 2:** Significant autoantibodies with high frequency differential in case and p-value <0.05.

Protein	Description	Penetrance frequency (case)	Penetrance frequency % (case)	Penetrance fold change (case)	Penetrance frequency (control)	Penetrance frequency % (control)	Penetrance fold change (control)	Frequency differential	Penetrance fold change difference	p-value	Log2 (overall fold change)
FKBP3	FK506 Binding Protein 3	6	20.00%	4.09	0	0.00%	0.00	6	4.09	0.023	0.73
TSPY3	Testis Specific Protein, Y-Linked 3	5	16.67%	4.82	1	3.33%	1.61	4	3.21	0.036	0.74
PTGER3	Prostaglandin E Receptor 3	4	13.33%	2.71	0	0.00%	0.00	4	2.71	0.014	0.37
NFIA	Nuclear Factor I/A	3	10.00%	5.63	0	0.00%	0.00	3	5.63	0.038	0.68
CXCR6	Chemokine (C-X-C Motif) Receptor 6	3	10.00%	3.93	0	0.00%	0.00	3	3.93	0.026	0.51
PDGFB	Platelet-Derived Growth Factor Beta Polypeptide	3	10.00%	3.04	0	0.00%	0.00	3	3.04	0.029	0.35
EIF4A3	Eukaryotic Translation Initiation Factor 4A3	3	10.00%	2.87	0	0.00%	0.00	3	2.87	0.012	0.44
MLKL	Mixed Lineage Kinase Domain-Like	3	10.00%	2.78	0	0.00%	0.00	3	2.78	0.011	0.39

**Table 3:** Significant autoantibodies with high frequency differential in control and p-value < 0.05.

Protein	Description	Penetrance frequency (case)	Penetrance frequency % (case)	Penetrance fold change (case)	Penetrance frequency (control)	Penetrance frequency % (control)	Penetrance fold change (control)	Frequency differential	Penetrance fold change difference	p-value	Log2 (overall fold change)
STUB1	STIP1 Homology And U-Box Containing Protein 1	0	0.00%	0.00	5	16.67%	8.17	-5	-8.17	0.043	-1.27
MAP4	Microtubule-Associated Protein 4	0	0.00%	0.00	5	16.67%	4.21	-5	-4.21	0.006	-0.78
STK33	Serine/Threonine Kinase 33	0	0.00%	0.00	4	13.33%	3.66	-4	-3.66	0.011	-0.58
USH1C	Usher Syndrome 1C	0	0.00%	0.00	3	10.00%	5.05	-3	-5.05	0.047	-0.63
CCNA2	Cyclin A2	0	0.00%	0.00	3	10.00%	3.36	-3	-3.36	0.023	-0.45

**Fig 7:**
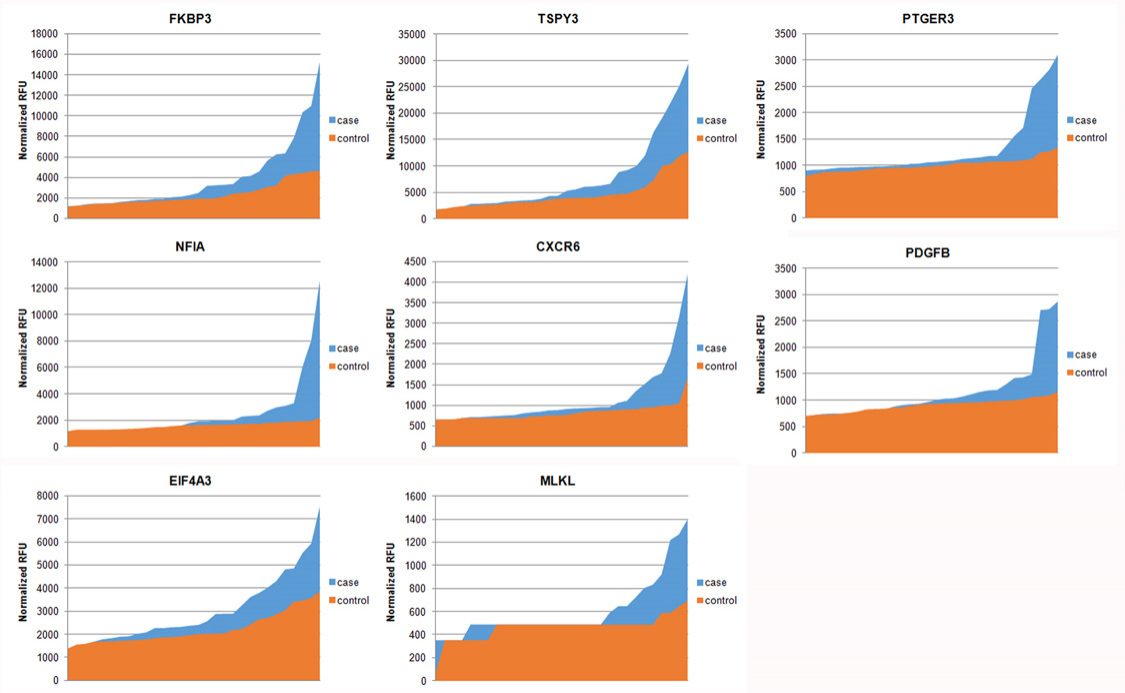
Normalized RFU (ranked from lowest to highest activities) between case and control groups for autoantibodies showing higher activity in the case group.

The volcano plot analysis is useful for the identification of significant autoantibodies. The volcano plot in Fig 6 shows differential-regulation of the autoantibodies listed in Tables 2 and 3. The up-regulated autoantibodies in case are located in the upper right side of the volcano plot (shown in red triangles) and down-regulated autoantibodies are located in the upper left part of the plot (shown in blue triangles). Ranked normalized RFU between case and control groups for each autoantibody are shown in Fig 7.

## Discussion

Whole cell antigen used in our serology test was prepared from a mix of five US *H*. *pylori* strains and previous studies have shown that antigenic differences exist among the *H*. *pylori* strains from different geographical regions [33,34]. To overcome the problem of poor antigenic recognition, two different antigens (WC and CagA) were used in this study. In a previous study in Ladakh, it was shown that as much as 27% of *H*. *pylori* culture-positive subjects responded to only the CagA antigen but not the WC antigen [33,34]. Thus, in this study, positive for WC and/ or CagA serology test was taken as *H*. *pylori* seropositive.

*H*. *pylori*-infected patients or mice immunized with the bacterium can develop autoantibodies that react with gastric mucosa of humans and mice, and murine monoclonal antibodies directed against *H*. *pylori* antigens can also react with gastric tissue [35–37]. Moreover, hybridoma secreting *H*. *pylori*-reactive monoclonal antibodies growing in mice caused histopathological changes similar to gastritis [35]. *H*. *pylori* CagA protein and platelet-associated IgG in *H*. *pylori*-associated chronic idiopathic thrombocytopenic purpura can cross react [38]. In addition, anti-CagA-seropositivity was associated with greater deterioration in parkinsonian facets with time [39]. Independent of anti-CagA, anti-nuclear antibody (ANA) has been associated with poor response to eradicating *H*. *pylori* in PD [40]. In addition, *H*. *pylori* colonization was associated with the release of large amounts of pro-inflammatory and vasoactive as interleukin (IL)-1β, -6, -8, -10, -13 and tumor necrosis factor (TNF)-α, eicosanoids, acute phase proteins and activated monocytes which may lead to disruption of blood brain barrier and microglial activation; with deleterious effect on the nigrostriatal dopaminergic system [24,41]. Taken together, these data suggest that the molecular mimicry between *H*. *pylori*, host antigens and *H*. *pylori*-induced autoantibodies may trigger an autoimmunity and chronic inflammatory state that contributed to the pathogenic process in gastric and extra-gastric diseases.

Among autoantibodies identified to be up-regulated in *H*. *pylori*-seropositive PD patients was nuclear factor I subtype A (NFIA), a member of the NFI/CAAT-box transcription factor. NFIA induce in mouse neurons by N-methyl-D-aspartate (NMDA) receptor activation in a nitric oxide synthase (NOS)- and Extracellular signal-regulated kinases (ERK)-dependent manner [42]. In the same study, using *Nfia*^*-/-*^ neurons and *Nfia*^*+/-*^ mice, NFIA was identified to be an important neuroprotective transcription factor in a complex survival mechanism that preconditions brain neuronal cells to endure toxic insults and that a deficiency of NFIA increases the susceptibility of neurons to injury [42].

Platelet-derived growth factors (PDGFs) are endogenous growth factors and the biologically active PDGF-BB dimer has been shown to have restorative effects in the dopaminergic system both *in vitro* and *in vivo* [43–47]. In a PD animal model study, it was demonstrated that intracerebroventricular (i.c.v) administration of PDGF-BB for 2 weeks restored dopamine transporter (DAT) binding and provided functional recovery [47]. Furthermore, the effect of PDGF-BB on dopaminergic neurons continued to develop after the treatment period and remained stable for several months after the end of treatment suggesting that the effects of PDGF-BB were mediated by proliferating periventricular progenitor cells [47]. Recently, a double-blind, randomized, placebo-controlled phase I/IIa study was carried out in Sweden to assess the safety and tolerability of i.c.v. recombinant human PDGF-BB (rhPDGF-BB) administration in PD subjects [48]. In the same study, all doses of i.c.v. administration of rhPDGF-BB were well tolerated and there was a positive effect on DAT binding to support further clinical development of rhPDGF-BB for patients with PD [48].

Another interesting finding from our result was the up-regulation of Eukaryotic translation initiation factor 4A3 (eIFA3) autoantibodies in PD patients who were *H*. *pylori* seropositive. *eIF4A3* mRNA is elevated in dorsal striatum and hippocampus following spatial exploration and is a key mediator of *Arc* mRNA availability underlying learning and memory processes in rats [49]. Although PD has been, until recently, mainly defined by the presence of characteristic motor symptoms, deterioration of the executive functions, such as attention, recognition, working memory, and problem solving, often appear in an early, premotor phase of the disease and progressively increase in intensity, negatively affecting the quality of life of ∼50%–60% of PD patients [50]. The cellular mechanisms underlying cognitive impairments in PD patients are largely unknown and an adequate treatment is still missing.

We also found other up-regulated antibodies (such as FKBP3, TSPY3, PTGER3, CXCR6, and MLKL) and down-regulated antibodies (such as STUB1, MAP4, STK33, USH1C and CCNA2) among the *H*. *pylori*-seropositive patients; however, the roles of these antibodies in PD and *H*. *pylori* remain unclear.

One of the limitations of this study is that only seroprevalence of *H*. *pylori* was determined, which does not distinguish current infections from previous exposure to the bacterium. In addition, the age of acquiring *H*. *pylori* and duration of colonization were not traceable. Whether patients were previously treated for *H*. *pylori* infection was also unknown. Such information may explain why only a subset of *H*. *pylori*-seropositive PD patients develops these autoantibodies. Most importantly, *H*. *pylori* isolates from these PD patients were not available for correlating of bacterial virulence factors and autoantibody levels.

In conclusion, preliminary data from the current retrospective study demonstrated that a subset of PD patients infected with *H*. *pylori* or previously exposed to the gastric pathogen develops elevated levels of autoantibody against host proteins. The immunomodulatary property of *H*. *pylori* may provide an explanation for the observation that *H*. *pylori* positivity was independently associated with greater PD motor severity [14]. However, further investigations are necessary to determine the role of these antibodies in PD pathogenesis and progression.

## Supporting Information

S1 Fig Plot represents the difference between raw median RFUs and background subtracted RFUs. (DOCX)

S2 Fig Plot represents average of each protein across all samples for both case and control groups. (DOCX)

S3 Fig Histogram plot showing normal distribution for 60 samples. (DOCX)

S4 Fig Boxplot of log2 (raw median RFU) and log2 (normalized median RFU). (DOCX)
