# Supplementary material for: Augmentation of Autoantibodies by Helicobacter pylori in Parkinson’s Disease Patients May Be Linked to Greater Severity
Source: PLoS One. 2016 Apr 21;11(4):e0153725. doi: 10.1371/journal.pone.0153725 (PMC4839651; doi:10.1371/journal.pone.0153725)
Supplement: S1 Fig — (DOCX) [file pone.0153725.s001.docx]

**Raw median RFU of replicates & background subtraction**

Raw median raw fluorescence unit (RFU) are the raw values derived from the array and in order to improve the quality of signal intensities, background RFUs for each spot were subtracted from raw values (Figure S1).

**Fig S1. Plot represents the difference between raw median RFUs (blue) and background subtracted RFUs (red).** The plot shows the average of the raw median RFU and background subtracted median RFU for each sample.
