# Supplementary material for: Augmentation of Autoantibodies by Helicobacter pylori in Parkinson’s Disease Patients May Be Linked to Greater Severity
Source: PLoS One. 2016 Apr 21;11(4):e0153725. doi: 10.1371/journal.pone.0153725 (PMC4839651; doi:10.1371/journal.pone.0153725)
Supplement: S2 Fig — (DOCX) [file pone.0153725.s002.docx]

**Plot of average of each protein across all samples**

**Fig S2. Plot represents average of each protein across all samples for both case and control groups.**
