# Supplementary material for: Augmentation of Autoantibodies by Helicobacter pylori in Parkinson’s Disease Patients May Be Linked to Greater Severity
Source: PLoS One. 2016 Apr 21;11(4):e0153725. doi: 10.1371/journal.pone.0153725 (PMC4839651; doi:10.1371/journal.pone.0153725)
Supplement: S3 Fig — (DOCX) [file pone.0153725.s003.docx]

**Histogram plot of log2 (median RFU)**

Histogram analysis of the log2 transformed raw data of 1631 proteins shows all protein data from 60 samples across all the proteins, including controls. This plot is useful to show spot variations and technical artifacts in the study. In this case, it shows clear normal distribution.

**Fig S3. Histogram plot showing normal distribution for 60 samples.**
