# Supplementary material for: Augmentation of Autoantibodies by Helicobacter pylori in Parkinson’s Disease Patients May Be Linked to Greater Severity
Source: PLoS One. 2016 Apr 21;11(4):e0153725. doi: 10.1371/journal.pone.0153725 (PMC4839651; doi:10.1371/journal.pone.0153725)
Supplement: S4 Fig — (DOCX) [file pone.0153725.s004.docx]

**Boxplot analysis**

The analysis of all protein data from the entire study shows that quantile normalization and normal transformation brought all the protein arrays onto similar scale. Normalization allows the exploration of outliers in the data and also highlights key changes occurring in the cohorts in order to shortlist putative biomarkers. It also increases the viability and precision of the protein signal intensities between cohorts which in turn affects the biomarker specificity.

**Fig S4. Boxplot of log2 (raw median RFU) (top) and log2 (normalized median RFU) (bottom).**
